# Effects of different thermal insulation methods on the nasopharyngeal temperature in patients undergoing laparoscopic hysterectomy: a prospective randomized controlled trial

**DOI:** 10.1186/s12871-021-01324-7

**Published:** 2021-04-05

**Authors:** Guanyu Yang, Zefei Zhu, Hongyu Zheng, Shifeng He, Wanyue Zhang, Zhentao Sun

**Affiliations:** Department of Anesthesiology, Pain and Perioperative Medicine, The First Affiliated Hospital of Zhengzhou University, Zhengzhou, Henan Province China

**Keywords:** Laparoscopic, Infusion thermometer, Warming blanket, Incubator, Nasopharyngeal temperature

## Abstract

**Background:**

This study explored the comparison of the thermal insulation effect of incubator to infusion thermometer in laparoscopic hysterectomy.

**Methods:**

We assigned 75 patients enrolled in the study randomly to three groups: Group A: Used warming blanket; group B: Used warming blanket and infusion thermometer; group C: Used warming blanket and incubator. The nasopharyngeal temperature at different time points during the operation served as the primary outcome.

**Results:**

The nasopharyngeal temperature of the infusion heating group was significantly higher than that of the incubator group 60 min from the beginning of surgery (T3): 36.10 ± 0.20 vs 35.81 ± 0.20 (*P*<0.001)90 min from the beginning of surgery (T4): 36.35 ± 0.20 vs 35.85 ± 0.17 (*P*<0.001). Besides, the nasopharyngeal temperature of the incubator group was significantly higher compared to that of the control group 60 min from the beginning of surgery (T3): 35.81 ± 0.20 vs 35.62 ± 0.18 (*P*<0.001); 90 min from the beginning of surgery (T4): 35.85 ± 0.17 vs 35.60 ± 0.17 (*P*<0.001). Regarding the wake-up time, that of the control group was significantly higher compared to the infusion heating group: 24 ± 4 vs 21 ± 4 (*P* = 0.004) and the incubator group: 24 ± 4 vs 22 ± 4 (*P* = 0.035).

**Conclusion:**

Warming blanket (38 °C) combined infusion thermometer (37 °C) provides better perioperative thermal insulation. Hospitals without an infusion thermometer can opt for an incubator as a substitute.

**Trial registration:**

This trial was registered with ChiCTR2000039162, 20 October 2020.

## Background

Reports describe perioperative hypothermia as a condition whereby the core body temperature drops below *36 °C* [1]*.* Its incidence rate ranges between 25 and 90% [2]. Of note, perioperative hypothermia is associated with a range of complications, such as intraoperative coagulation dysfunction, delayed postoperative recovery, incision infection, among others [3, 4].

Laparoscopic surgery presents benefits, including less trauma, less bleeding, more rapid postoperative recovery, fewer surgical complications, etc. [5, 6]. Compared to open surgeries, the abdominal cavity is relatively more closed, however, anesthetic factors, persistent C0_2_ pneumoperitoneum during surgery, and the utilization of huge amounts of irrigating fluid may induce hypothermia [7, 8].

In this study, our main focus was to compare the effects of two different thermal insulation methods in laparoscopic hysterectomy.

## Methods

Approval for this prospective, single-blind, randomized, controlled study was issued by the Ethics Committee of the First Affiliated Hospital of Zhengzhou University (2020-KY-176), registered at http://www.chictr.org.cn/index.aspx (ChiCTR2000039162) on 20 October 2020. All patients signed informed consent. The protocol for this work strictly conformed with the international guidelines for randomized clinical studies and the CONSORT Guidelines.

We scheduled 75 patients for elective laparoscopic hysterectomy. Inclusion criteria included:1) Patients aged 40–65 years; 2) patients classified in the American Society of Anesthesiologists’ physical status class of I to II; 3) patients without severe heart, liver, kidney disease; 4) patients with no history of severe respiratory or cerebrovascular disease. Exclusion criteria included: 1) preoperative anemia; 2) intraoperative blood transfusion; 3) transition to open surgery. Using a random number table, we randomly divided the 75 patients into 3 groups: The Control group (A), infusion heating group (B), and incubator group (C).

### Study protocol

Premedication was not given to any patient. The operating room was maintained at 22–24 °C. All three groups were provided with warming blankets, which they turned on an hour in advance to achieve the preset temperature (38 °C). Upon entry to the operating room, we first administered the patients in the three groups with an injection of 500 ml Ringer’s lactate solution and followed by 500 ml succinylated *gelatin.* The infusion speed was maintained at 10 ml/min. Notably, in cases where the above liquids were both infused and replaced with 100 ml Ringer’s lactate solution, we ensured that the infusion path was clear and the infusion speed was nearly stopped. In group A, the infusion fluid was not treated and maintained at room temperature; in group B, we heated the infusion fluid by the infusion thermometer, and set the target temperature of the infusion thermometer 37 °C; in group C, the infusion fluid was incubated with the set target temperature at 37 °C.

ECG, BP, HR, SpO_2_, and BIS were monitored in all patients after they got into the operating room. Sufentanil 0.5 μg/kg, cisatracurium 0.25 mg/kg and etomidate 0.2 mg/kg were intravenously administered as anesthetic induction. Following the insertion of the laryngeal mask, the ventilator was set at V_T_ 6 ~ 8 ml /kg, FiO_2_50%, I: E 1: 2, RR12 ~ 20 times/min. P_ET_CO_2_ was maintained at 35 ~ 45 mmHg. Anesthesia was maintained with sevoflurane (gas flow at 2 L/min), remifentanil, and cisatracurium. Sevoflurane was titrated to maintain BIS of 40 to 60 during surgery.

The nasopharyngeal temperature was assessed following the induction of anesthesia in the three groups. Briefly, the nasopharyngeal probe was inserted to of about 1 cm depth beyond the first scale (10 cm). It was then secured with tape to maintain the depth. Vital signs were stabilized during the operation. At lower intraoperative nasopharyngeal temperature below 35 °C, the temperature of the warming blanket was raised to maintain the patient’s nasopharyngeal temperature above 35 °C.

### Outcome measurements

In the three groups, the primary outcome was the nasopharyngeal temperature at 5 min post anesthesia induction (T1), 30 min (T2), 60 min (T3), and 90 min (T4) at the beginning of surgery, whereas the secondary outcome was wake-up time.

### Statistical analyses

SPSS (version 22.0, SPSS Inc., Chicago, IL, USA) was applied to analyze all statistical data. Measurement data were expressed as mean ± standard deviation. We adopted a one-way analysis of variance for comparison between groups; the nasopharyngeal temperature was compared at different time points via repeated measurement ANOVA. For counting data, the Chi-square test was used for comparison. *P* < 0.05 denoted statistical significance.

The sample size was established using GPower (version 3.1.9.2, Franz Faul, Universitat Kiel, Germany). Reports have demonstrated that a core temperature difference of 0.5 °C is clinically significant; it is the smallest difference associated with hypothermic complications [9]. With the significance level(α) set at 0.05, and power(1-β) at 0.9, each group should include 22 patients, assuming that the withdrawal rate is 10%. Eventually, each group comprises 25 patients.

## Results

**Fig. 1:**
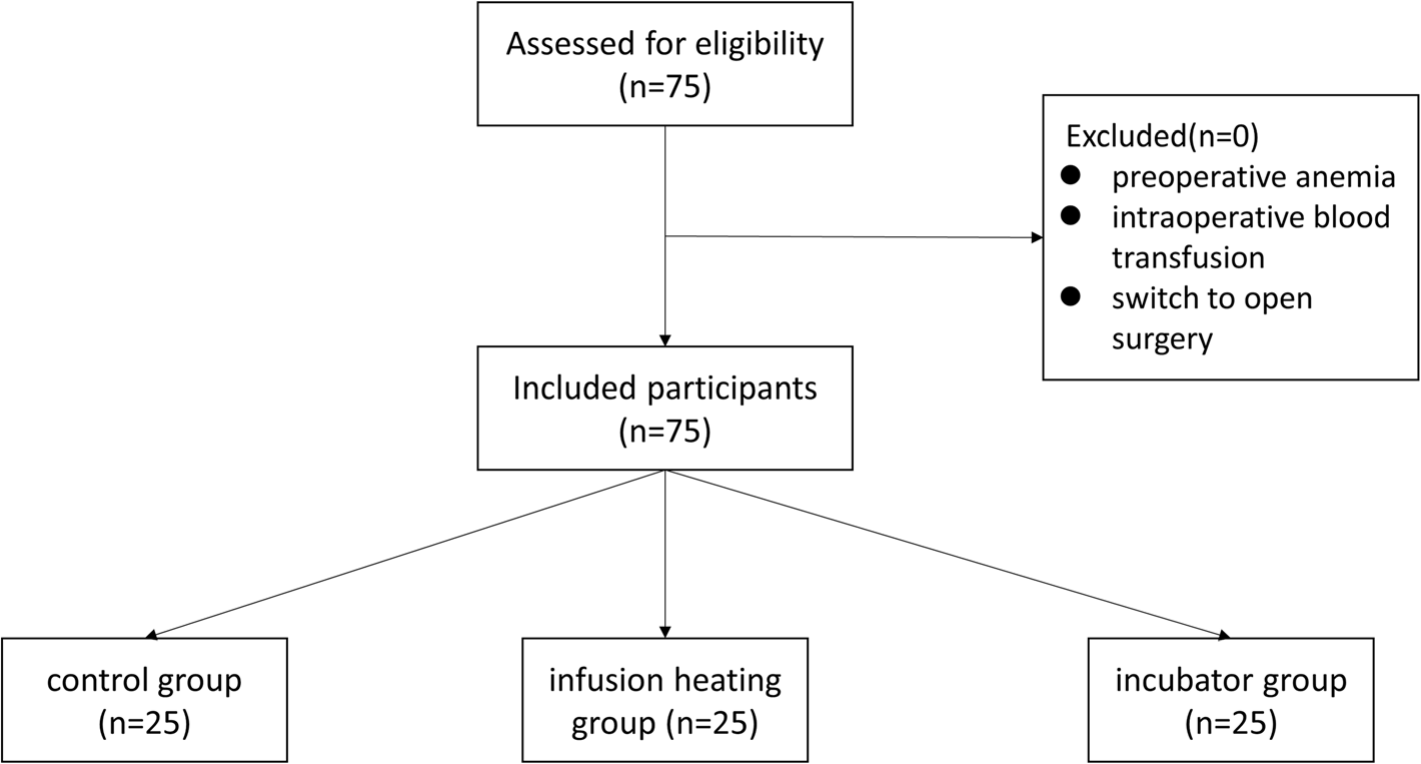
Participant flowchart

Of the 75 enrolled patients (Fig. 1), none exhibited intraoperative nasopharyngeal temperature below 35 °C.

### Primary outcome

**Fig. 2:**
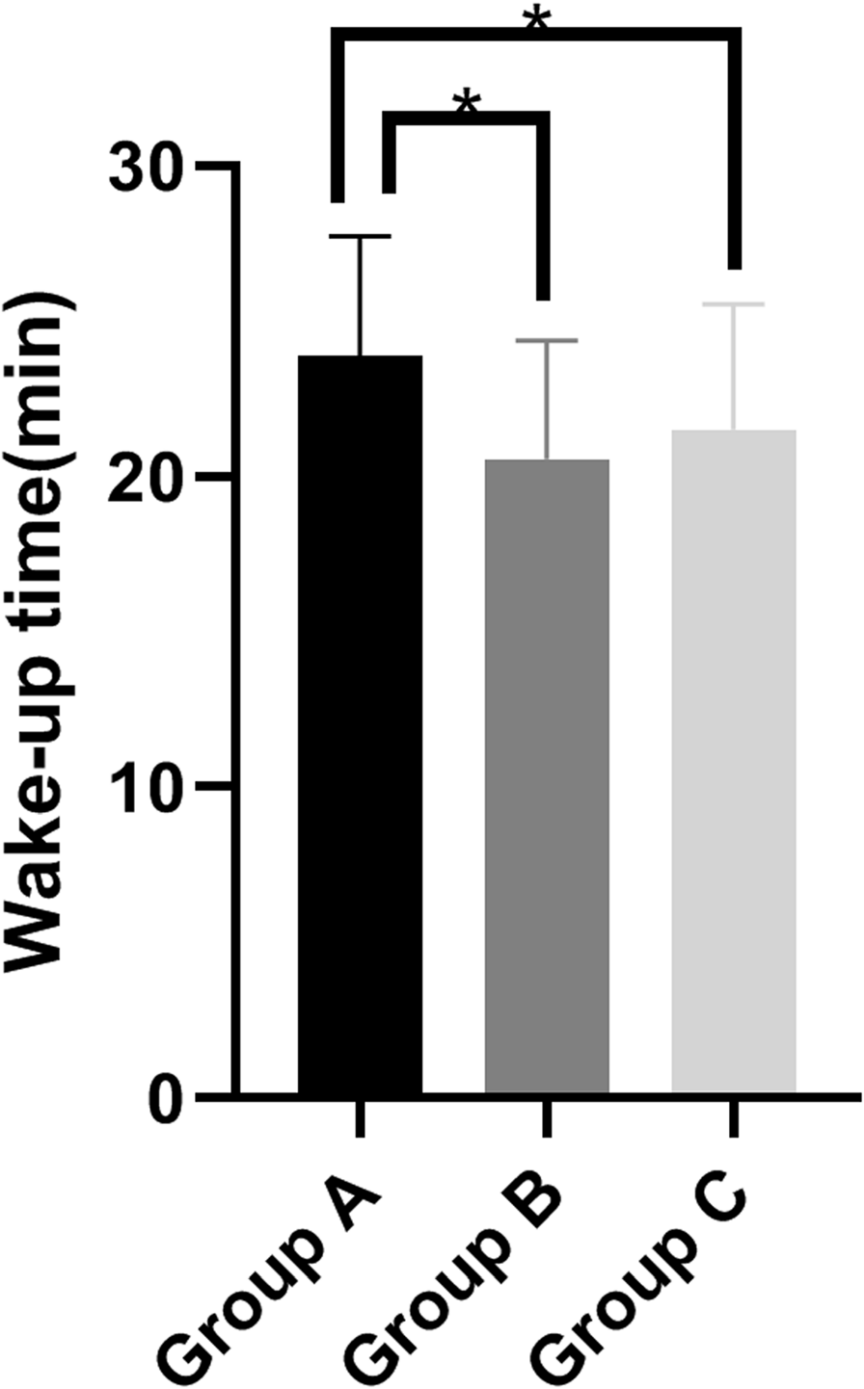
Nasopharyngeal temperature at different points in three groups. Time point, 5 min after anesthesia induction (T1), 30 min at the beginning of surgery (T2), 60 min at the beginning of surgery (T3), 90 min at the beginning of surgery (T4); ^+^statistical significance between the group A and group B; ^*^statistical significance between the group A and group C; ^#^statistical significance between the group B and group C

Compared to the incubator group, the nasopharyngeal temperature of the infusion heating group was significantly higher at 60 min from the beginning of surgery (T3): 36.10 ± 0.20 vs 35.81 ± 0.20 (*P*<0.001); 90 min from the beginning of surgery (T4): 36.35 ± 0.20 vs 35.85 ± 0.17 (*P*<0.001). Besides, the nasopharyngeal temperature of the incubator group was significantly higher than that of the control group 60 min from the beginning of surgery (T3): 35.81 ± 0.20 vs 35.62 ± 0.18 (*P*<0.001); 90 min from the beginning of surgery (T4): 35.85 ± 0.17 vs 35.60 ± 0.17 (*P*<0.001)(Table 1)(Fig. 2).

**Table 1:** Nasopharyngeal temperature at different points in three groups

Group	n	T1	T2	T3	T4
A	25	36.40 ± 0.19	35.88 ± 0.21^t^	35.62 ± 0.18^t^	35.60 ± 0.17^t^
B	25	36.37 ± 0.24	35.92 ± 0.24^t^	36.10 ± 0.20^*t^	36.35 ± 0.20^*^
C	25	36.35 ± 0.21	35.88 ± 0.18^t^	35.81 ± 0.20^*#t^	35.85 ± 0.17^*#t^

Values are presented as mean ± standard deviation

5 min after anesthesia induction (T1), 30 min at the beginning of surgery (T2), 60 min at the beginning of surgery (T3), 90 min at the beginning of surgery (T4)

^*^P<0.05 compared to the same time of Group A

^#^*P*<0.05 compared to the same time of Group B

^t^*P*<0.05 compared to T1

### Secondary outcomes

**Fig. 3:**
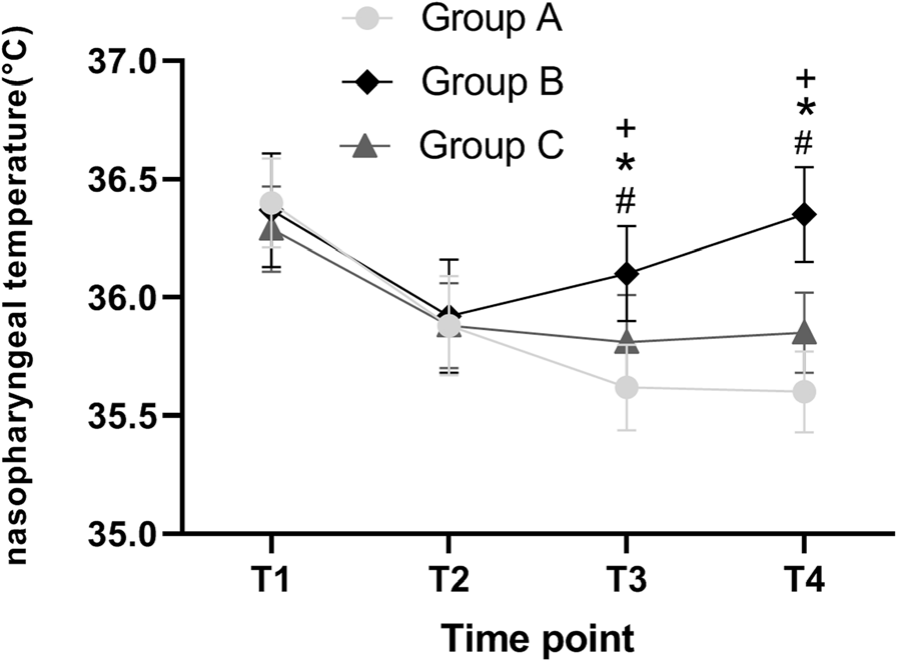
Wake-up time in three groups; ^*^P<0.05

The wake-up time of the control group was significantly higher than that of the infusion heating group: 24 ± 4 vs 21 ± 4 (*P* = 0.004) and the incubator group: 24 ± 4 vs 22 ± 4 (*P* = 0.035) (Table 2) (Fig. 3).

**Table 2:** Descriptive variables of the group A, group B and group C

	Group A(*n* = 25)	Group B(*n* = 25)	Group C(*n* = 25)	F value	*P* value
Age (years)	53.28 ± 6.91	51.52 ± 7.6	54.32 ± 6.30	1.034	0.361
BMI (kg/m^2^)	23.31 ± 3.07	23.50 ± 2.91	23.62 ± 3.01	0.066	0.936
ASA grade(I/II)	13/12	16/9	12/13	/	0.497
Surgery time (min)	108 ± 8	111 ± 8	109 ± 9	0.770	0.467
Anesthesia time (min)	135 ± 9	139 ± 8	136 ± 9	1.279	0.284
Blood loss (ml)	38.60 ± 6.70	39.80 ± 6.69	40.28 ± 8.84	0.335	0.717
Urine output (ml)	338.00 ± 96.05	340.00 ± 76.38	358.00 ± 89.77	0.394	0.676
Wake-up time (min)^a^	24 ± 4	21 ± 4	22 ± 4	4.816	0.011
Infusion volume (ml)	1000	1000	1000	/	/

Values are presented as mean (standard deviation) or counts

*ASA* American society of anesthesiologists, *BMI* Body Mass Index

^a^Wake-up time: The time from the patient stops inhaling sevoflurane until the laryngeal mask was removed

## Discussion

The present findings demonstrated that nasopharyngeal temperature decreased significantly in the three groups from T1 to T2. We revealed that general anesthesia induces peripheral vasodilation, which impedes the function of blood vessels to regulate the body temperature via contraction. Meanwhile, general anesthesia inhibits central *thermoregulation of the body*, whereas anesthetic drugs lower the metabolic rate [10, 11]. At relatively low operating room temperature, a lot of heat is absorbed by preoperative disinfection and intraoperative infusion; some heat is propelled by continuous CO_2_ infusion during the operation [8, 12].

Herein, the nasopharyngeal temperature of the T2 to the T3 control group and the incubator group continuously decreased. Notably, the degree of decrease in the incubator group was less compared to that of the control group. The rise in the nasopharyngeal temperature of the infusion heating group demonstrated that the use of a warming blanket alone (38 °C) was inefficient to maintain the patient’s body temperature. Maybe it needed a higher temperature. Furthermore, the infusion fluid in the incubator group was not continuously heated. In consequence, the temperature gradually decreased over time. It is solely in the infusion heating group whereby the rise in the patient’s body temperature was induced by a warming blanket and infusion thermometer.

As the nasopharyngeal temperature of the T3 to T4 control group started to stabilize, the nasopharyngeal temperature of the incubator group began to rise, though lower than T1. The nasopharyngeal temperature of the infusion heating group continuously rose, with no significant difference from T1. We deduced that the temperature of the patients was potentially maintained at the preoperative level when the warming blanket (38 °C) combined infusion thermometer (37 °C) was used for 90 min.

Moreover, the wake-up time of the control group was significantly higher compared to that of the infusion heating group and the incubator group. This was primarily attributed to the low-temperature status, which lowered the uptake capacity of the liver for drugs and the kidney capacity to excrete drugs. Consequently, there arises an impact on the metabolism of anesthetic drugs in the body which is potentially associated with prolonged wake-up time [13, 14].

As one of the vital signs, body temperature has increasingly been attractive in the perioperative period in recent years. However, the maintenance of body temperature during the perioperative period is unsatisfactory. Of note, this may be linked to the insufficient attention paid by medical staff to maintain body temperature. Besides, there is no protective device for body temperature in hospitals, for example, an infusion thermometer [15, 16]. Usually, the incubator is an essential equipment for hospitals to preserve the irrigating fluid used during the operation. The present study fully utilized the equipment to preserve the injected fluid during the operation. This aimed to achieve the effect of fluid heating. Although the patient experienced hypothermia during the operation, the temperature drop was minimal. Also, the wake-up time was short. We, therefore, recommend this method for hospitals without a better insulation device, owing to its straightforward use, low cost and we expect this method to be popularized in clinical practice.

This work is limited to the observation time which was restricted to 90 min. Thus, for a longer duration of surgery, it remains unclear whether the temperature of the warming blanket and infusion thermometer should be adjusted, which warrants further exploration.

## Conclusions

Warming blanket (38 °C) combined infusion thermometer (37 °C) provides better perioperative thermal insulation. Notably, hospitals without an infusion thermometer may still opt for an incubator as a substitute.

## Data Availability

The raw data of this study are available from the corresponding author on reasonable request.

## Abbreviations

BMI: Body Mass Index
ASA: American Society of Anesthesiologists
ECG: Electrocardiogram
BP: Blood Pressure
HR: Heart Rate
SpO_2_: Pulse Oxygen Saturation
BIS: Bispectral Index
